# Significance and Applications of the Thermo-Acidophilic Microalga *Galdieria sulphuraria* (Cyanidiophytina, Rhodophyta)

**DOI:** 10.3390/plants13131786

**Published:** 2024-06-27

**Authors:** Berhan Retta, Manuela Iovinella, Claudia Ciniglia

**Affiliations:** 1 Department of Engineering, University of Campania Luigi Vanvitelli, Via Roma 29, 81031 Aversa, Italy; berhan.retta@unicampania.it; 2 Department of Environmental, Biological and Pharmaceutical Sciences and Technologies, University of Campania Luigi Vanvitelli, Via Vivaldi 43, 81100 Caserta, Italy; manuela.iovinella@unicampania.it

**Keywords:** *Galdieria sulphuraria*, bioactive compounds, wastewater, metals

## Abstract

*Galdieria sulphuraria* is a thermo-acidophilic microalga belonging to the Cyanidiophyceae (Rhodophyta) class. It thrives in extreme environments, such as geothermal sulphuric springs, with low pH, high temperatures, and high salinity. This microalga utilises various growth modes, including autotrophic, heterotrophic, and mixotrophic, enabling it to exploit diverse organic carbon sources. Remarkably, *G. sulphuraria* survives and produces a range of bioactive compounds in these harsh conditions. Moreover, it plays a significant role in environmental remediation by removing nutrients, pathogens, and heavy metals from various wastewater sources. It can also recover rare earth elements from mining wastewater and electronic waste. This review article explores the diverse applications and significant contributions of *G. sulphuraria*.

## 1. Introduction

**Figure 1:**
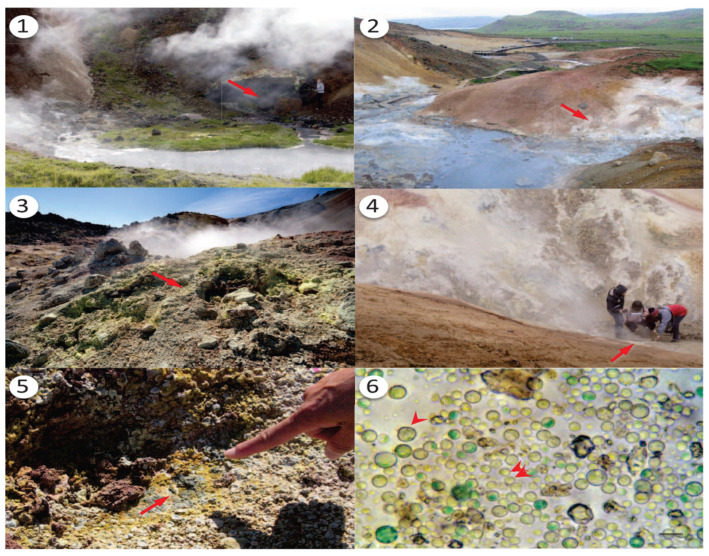
Five Icelandic geothermal regions for the Cyanidiophyceae. Arrows indicate the collection sites from the stations. 1. Nesjavellir of Thingvellir, southwestern Iceland. 2. Seltun of Krisuvik, southwestern Iceland. 3. Landmannalaugar of Hekla, southeastern Iceland. 4. Viti of Krafla, northeastern Iceland. 5. Closer look at the Landmannalaugar collection site. 6. Light microscopic image of the Cyanidiophyceae collected from Landmannalaugar [17].

Microalgae, photosynthetic microorganisms, use carbon dioxide to generate organic matter and release oxygen. They produce valuable compounds, such as carbohydrates, lipids, and bioactive substances [1], and are excellent sources of fatty acids and antioxidants [2]. Additionally, microalgae play significant roles in wastewater treatment and carbon dioxide mitigation [3]. *G. sulphuraria* is a unicellular thermo-acidophilic microalga belonging to the class Cyanidiophyceae [4,5,6,7]. It thrives in some of the most extreme environments known for eukaryotic organisms (Figure 1(1–6)), mainly inhabiting geothermal sulphuric springs and other hostile settings characterised by extreme acidity, high temperatures, darkness, and high concentrations of salts, arsenic, and toxic metals [8]. This extremophilic alga exhibits remarkable versatility in its growth modes, including photo-autotrophy, photo-heterotrophy, and chemo-heterotrophy [9], allowing it to exploit various organic carbon sources efficiently. Despite these harsh environmental conditions, *G. sulphuraria* demonstrates exceptional resilience. It can withstand a pH range of 0.05 to 4, temperatures as high as 56 °C, and substantial salt concentrations [8,10,11,12,13]. This resilience makes it a valuable organism for scientific research and practical applications. *Galdieria*’s unique adaptations enable it to play a significant role in various biotechnological applications [14]. It has been extensively studied for its potential in wastewater treatment, where it aids in removing heavy metals and other contaminants [15]. Furthermore, *G. sulphuraria* is instrumental in recovering rare earth elements from mining wastewater and electronic waste, contributing to resource recycling and environmental sustainability [16]. Additionally, it is known for synthesising a range of bioactive compounds, which have potential uses in pharmaceuticals, nutraceuticals, and other industries.

This review article aims to comprehensively document the research findings related to *G. sulphuraria*, mainly focusing on its applications in wastewater treatment, heavy metal removal, rare earth element recovery, and the synthesis of valuable bioactive compounds. By compiling and analysing these insights, this article seeks to highlight *Galdieria*’s potential in addressing some of our time’s critical environmental and industrial challenges.

## 2. Synthesis of Bioactive Compounds

*G. sulphuraria* can produce various bioactive compounds with significant potential across multiple biotechnological fields (Table 1). One of the most notable groups of these compounds is phycobiliproteins. These fluorescent proteins are located in phycobilisomes, auxiliary photosynthetic complexes that enhance energy capture during photosynthesis. Phycobiliproteins have gained considerable attention due to their antioxidant, antibacterial, and antitumor properties, rendering them valuable in biomedicine, bioenergy, and scientific research [18]. Phycobiliproteins consist of three primary types: C-phycocyanin, phycoerythrin, and allophycocyanin [19]. Each protein contributes uniquely to the alga’s functionality and potential applications; C-phycocyanin is a blue pigment–protein complex widely used in the food and cosmetic industries as a natural dye [20,21]. Moreover, it exhibits potent antioxidant properties, which help neutralise free radicals and reduce oxidative stress [22]. Its anti-inflammatory and neuroprotective effects have also made it a focus of medical research for potential therapeutic applications [23,24].

Phycoerythrin, known for its bright red colour, is highly efficient in capturing light energy, which makes it useful in various photobiological applications. Due to its strong fluorescence and stability, it is extensively utilised in fluorescence-based techniques such as flow cytometry and fluorescence microscopy [25]. Additionally, its antioxidant and anti-inflammatory properties add value to biomedical research and applications.

Allophycocyanin protein is an intermediate energy transfer pigment within the phycobilisome complex [21]. Allophycocyanin’s fluorescence properties make it a valuable tool in scientific research, particularly in molecular biology techniques where it is used as a fluorescent marker. Its role in enhancing the overall efficiency of the photosynthetic process also underscores its potential in bioenergy applications, where improving photosynthetic efficiency is a crucial goal.

**Table 1:** Bioactive compounds produced by *G. sulphuraria*.

Bioactive Compounds	Uses	References
Phycobiliproteins	Antioxidant, antibacterial, and antitumor properties	[18]
C-phycocyanin	A natural dye in the food and cosmetic industries Exhibits potent antioxidant properties Anti-inflammatory and neuroprotective effects	[19] [22,26,27,28] [23,24]
Phycoerythrin	Utilized in fluorescence-based techniques (flow cytometry and fluorescence microscopy)	[25]
Allophycocyanin	Used as a fluorescent marker	[21]
Glutathione	Antioxidant–protects cells from oxidative stress, maintains cellular redox balance; Involved in detoxification, immune function, and regulation of cellular proliferation and apoptosis	[29,30,31] [30,32,33]

Beyond phycobiliproteins, *G. sulphuraria* produces other bioactive compounds, including various polysaccharides, lipids, and secondary metabolites. These compounds have applications ranging from developing biofuels and biodegradable materials to formulating pharmaceuticals and nutraceuticals.

### 2.1. Synthesis of Phycocyanin (PC)

Phycocyanin, one of the components of phycobiliprotein, has a blue colour and water-soluble property. It is predominantly present in cyanobacteria and Rhodophyta [34,35]. It has usages in different industries, such as cosmetics, diagnostics, foods, and as a nutraceutical or biopharmaceutical [28,35,36]. C-phycocyanin (C-PC) represents the major light-harvesting biliprotein, known for its central role in absorbing light [26]. C-phycocyanin is a potent antioxidant that helps scavenge free radicals and reduces oxidative stress in cells [37]. This property is beneficial in protecting cells from damage, which is crucial for preventing chronic diseases and ageing. As an anti-inflammatory compound, C-phycocyanin can inhibit the production of pro-inflammatory cytokines, making it helpful in treating inflammatory conditions [24]. Research indicates that C-phycocyanin may also have neuroprotective effects, helping to protect nerve cells from damage and potentially offering benefits in neurodegenerative diseases, such as Alzheimer’s and Parkinson’s [38]. Potential anticancer activity by inducing apoptosis (programmed cell death) in cancer cells and inhibiting tumour growth is of great interest; its ability to selectively target cancer cells while sparing normal cells makes it a promising candidate for cancer therapy [39].

The market and economic value of phycocyanin have seen significant growth in recent years. Phycocyanin is expected to reach a market value of USD 409.8 million by 2030 [40].

C-PC from heterotrophic *G. sulphuraria* has comparable properties to cyanobacterial C-PC and can be purified to the same standards [36]. Similarly, *G. sulphuraria* was shown to produce more thermostable phycocyanin than *Spirulina platensis* [41]. Additionally, better *G. sulphuraria* C-PC stability as compared to C-PC obtained from *S. platensis* at temperatures between 50 and 65 °C and in a neutral environment of pH 7 was documented. Also, a higher mid-unfolding temperature (73 °C vs. 69.81 °C) and similar antioxidant capacity as *S. platensis* were reported [42].

Rahman et al. [43] obtained thermally stable phycocyanin from *Galdieria* sp. 009, which can be used as an alternative to *Spirulina* phycocyanin (>47 °C) for natural blue food colouring. The authors reported the highest phycocyanin content by doubling ammonium sulphate in Allen medium (100 mg/g). Even though high content was obtained during extraction at pH 7, superior thermostability (>60 °C) and purity were found at pH 5. Thermostable phycocyanin from *G. sulphuraria* was extracted using freeze–thaw cycles, and the purification was performed with ammonium sulphate fractionation, which resulted in phycocyanin with a high purity ratio (A620/A280 > 4) and maximum absorbance at 620 nm [44]. The authors stated a recovery efficiency of >80% and a 19 mg pure phycocyanin yield from 3 g of *Galdieria* sp. wet cell mass.

The effect of light and nitrogen on phycocyanin production in *G. sulphuraria* 074G was investigated, and the batch cultures at the exponential growth phases gave different phycocyanin. Reports of 2–4 mg PC per g dry weight in carbon-limited and nitrogen-sufficient batch cultures grown in darkness and 8–12 mg PC/g dry weight during the stationary phase have been made, whereas the phycocyanin content in nitrogen-deficient cells decreased to values below 1 mg/g dry weight during stationary phase. In mixotrophic cultures, there was no light effect with glucose/fructose but increased phycocyanin in a glycerol-grown culture, from 10 mg/g in darkness to 20 mg/g at 80 µmol photons m^2^/s. The highest steady-state PC content (15–28 mg/g) was obtained at 65 µmol photons m^2^/s under continuous flow culture on glucose/glycerol. Regardless of the low PC production, *Galdieria*’s ability to produce PC in different heterotrophic or mixotrophic conditions indicates its potential as an alternative to *S. platensis* for PC production [45]. *G. sulphuraria* was cultivated using the ‘Sequential Heterotrophy-Dilution-Photoinduction’ strategy, and high phycocyanin (up to 13.88% of their dry cell weight) was obtained. This value was compared with the literature values of *G. sulphuraria* and was found to be 147-fold and 12-fold of those in photoautotrophic and heterotrophic technologies, respectively [46].

#### Eco-Friendly Production of C-phycocyanin by Cultivating *Galdieria* on Food Waste

The growing demand for sustainable and eco-friendly production methods in biotechnology has led to innovative approaches to utilising waste materials. One such approach involves cultivating *G. sulphuraria* in food waste to produce C-phycocyanin. This method addresses the issue of food waste disposal and provides a sustainable way to produce valuable bioactive compounds.

*G. sulphuraria* was allowed to grow both in an optimised tangerine peel and glucose media, and the amount of phycocyanin was compared, in which the tangerine peel medium showed a 1.7-fold increase in phycocyanin yield (159 mg/L) compared to the glucose medium (93.67 mg/L) [47]. The biomass yield of most microalgae is limited because of their autotrophic nature, but this issue can be overcome by using a heterotrophic growth mode with suitable carbon sources and oxygen. *G. sulphuraria* strain 074G grew on maltodextrins and granular starch in combination with the enzyme cocktail Stargen002. The result indicated that maltodextrin cultures produced 2 mg phycocyanin per gram substrate, slightly more than the yield from glucose. Phycocyanin from maltodextrin-grown cultures was thermostable up to 55 °C [48]. Portillo et al. [49] showed that PC production of *G. sulphuraria* UTEX 2919 under heterotrophic growth conditions is related to the carbon source, whereby the highest PC accumulation was reached in the presence of glucose (165 mg PC/g of soluble protein) and glucose enzymatic hydrolysate of corn stove (180 mg PC/g of soluble protein).

Biomass and phycocyanin production in highly pigmented variants of *G. sulphuraria* was also investigated [50]. These variants maintained high specific pigment concentrations when grown heterotrophically in darkness. Cultures reached biomass concentrations of 80–110 g/L and PC concentrations of 1.4–2.9 g/L, with volumetric PC production rates ranging from 0.5 to 0.9 g/L/day. Notably, the PC production rate was 11–21-times higher than those previously reported for heterotrophic *G. sulphuraria* 074G grown on glucose and 20–287-times higher than in phototrophic cultures of *S. platensis*, the organism used for commercial PC production. In another study, *G. sulphuraria* at a photon fluence rate of 200 μmol photons m^2^/s attained a growth rate of 10 g/m^2^/day, 33-day maximal biomass of 232 g/m^2^, and phycobilin content of 14 g/m^2^ in the biomass of 63 mg/g at a photon fluence rate of 100 μmol photons m^2^/s [19].

Amylolytic and proteolytic hydrolysed food waste from restaurants and bakeries was used to grow *G. sulphuraria* 074G heterotrophically. This process used carbohydrates and amino acids from the waste, requiring ammonium and inorganic nutrients for phycocyanin synthesis. At temperatures of 25 °C or 34 °C, the highest phycocyanin contents (20–22 mg/g) were observed on food wastes. Growth inhibition was observed when the hydrolysates were used in quantities resulting in glucose concentrations of 10 and 50 g/L growth inhibition for bakery and restaurant waste, respectively [51].

*G. sulphuraria* ACUF 064 was grown in a 13 L lab-scale photobioreactor under mixotrophic and heterotrophic conditions, using buttermilk as a carbon source. The result presented a 70% higher biomass yield in mixotrophic than heterotrophic conditions for galactose and lactose, but the same for glucose. A biomass of 0.55 g/L/d, carbon removal of 61%, and a high level of C-phycocyanin (5.9% w_C-PC/_w_x_) were indicated under mixotrophic conditions [52].

### 2.2. Antioxidant Activity of Galdieria sulphuraria

The antioxidant properties of biomass extracted from *G. sulphuraria* and *G. phlegrea* have been well-documented, primarily due to their high levels of phycocyanin and glutathione [26,53,54]. Bottone et al. [26] reported that cells extracted from heterotrophic *G. sulphuraria* demonstrated significant antioxidant capabilities and exhibited cytotoxic effects on the human adenocarcinoma cell line A549. Further studies by Massa et al. [27] have revealed that *G. sulphuraria* grown in spent cherry-brine liquid (sCBL) exhibited higher antioxidant activity and increased carbohydrate and polyphenol content, indicating the growing medium’s influence on the antioxidant potential of *G. sulphuraria.* This indicates that the growing medium can influence the antioxidant potential of *G. sulphuraria.* Moreover, a comparative study by Gürlek et al. [28] assessed the antioxidant activities of crude extracts from different microalgae species after 48 h exposure to human hepatocellular liver carcinoma cells (HepG2). *G. sulphuraria* showed the highest radical scavenging activity (95% RSA) and had a phenolic content of 312 mg GAE (Gallic Acid Equivalent) per mg of extract.

Glutathione (GSH) is a tripeptide composed of three amino acids: glutamine, cysteine, and glycine [54,55,56]. It is a critical antioxidant in cellular defence mechanisms, playing a vital role in protecting cells from oxidative stress and maintaining cellular redox balance [29,30]. Glutathione is involved in various biochemical processes, including detoxification, immune function, and regulation of cellular proliferation and apoptosis [30,32,33]. *G. sulphuraria* has gained attention for its robust antioxidant capabilities, partly attributed to its high glutathione content, contributing to its resilience in extreme environments [31]. The high intracellular levels of glutathione help protect the alga from oxidative damage caused by reactive oxygen species (ROS) generated under stressful conditions, such as high temperature, low pH, and the presence of toxic metals. Moreover, by maintaining the redox balance and supporting various cellular functions, glutathione helps ensure the overall health and metabolic efficiency of *G. sulphuraria* [31], contributing to its productivity and robustness as a biotechnological resource. Glutathione also plays a role in detoxifying harmful substances, including heavy metals and xenobiotics, through conjugation reactions. This detoxification capability enhances the potential of *G. sulphuraria* in bioremediation applications, such as wastewater treatment and the recovery of valuable elements from polluted sources.

### 2.3. Nutritional Properties of Galdieria sulphuraria

The storage glucans of *G. sulphuraria* were reported to be glycogen [57], which can be utilised in sports beverages and peritoneal dialysis solutions [4]. *G. sulphuraria* was reported to accumulate small, highly branched glycogen (7–18%) in large amounts, which could represent an excellent alternative to starch as a substrate for producing highly branched glucose polymers [58]. According to the authors, this glycogen synthesised by *Galdieria* is less susceptible to digestive enzymes and has less viscosity in solution than starch-derived polymers. Moreover, a highly branched glycogen was reported to have been synthesised by *G. sulphuraria* under heterotrophic conditions [59]. The authors showed that this glycogen had 18% of α-(1 → 6) linkages, short chains, and a small molecular weight and particle size compared to other glycogens.

Sakurai et al. [60] also investigated *G. sulphuraria* and revealed that mixotrophic cultures produced the highest biomass and increased glycogen levels, while neutral lipid amounts were similar to heterotrophic cultures. According to the authors, glycogen structure and fatty acid composition were influenced by growth conditions. Floridoside, another type of carbohydrate with antifouling and therapeutic properties, can be synthesised by *G. sulphuraria* [61]. These authors reported a maximum yield of 56.8 mg/g dry biomass, using glycerol as a carbon source.

### 2.4. Galdieria sulphuraria as a Food Source

There are a few published research results that recommended *G. sulphuraria* as a food source; Graziani et al. [62], for instance, reported that heterotrophic cultivation of *G. sulphuraria* showed high protein (26–32%) and polysaccharide (63–69%) content, low lipids, and hence recommended *G. sulphuraria* biomass as a food ingredient. Moreover, Abiusi et al. [63] explored *G. sulphuraria* as a protein source. Four strains showed varying amino acid profiles but met FAO dietary requirements for adults. The specific growth rates ranged from 1.01 to 1.48/day. After glucose depletion, the nitrogen content rose by 38–49% within 48 h, reaching 7.8–12.0% (*w*/*w*). Protein bioaccessibility decreased from 69% in exponential growth to 32% 48 h post-stationary phase. Selecting the correct strain and harvest time is the key to effective single-cell protein production. Additionally, Abiusi et al. [64] reported no significant difference in C-phycocyanin and protein contents between autotrophic and ‘oxygen balanced’ mixotrophic cultivations of *G. sulphuraria* in a chemostat. However, the mixotrophy showed doubled biomass productivity and concentration. A stable C-PC and protein content (62% *w*/*w*) of *G. sulphuraria* that meets FAO recommendations was reported as a promising candidate for food and feed application, and hence large-scale, efficient cultivation using oxygen-balanced mixotrophy was highlighted. Similarly, *G. sulphuraria* potential in food applications was assessed at a low pH (<1.9) using two strains: SAG 108.79 and ACUF 064. The autotrophic and mixotrophic biomass productivity of both strains was similar. However, their protein and C-phycocyanin content showed variations; 51% and 64% protein and 4% and 9% C-phycocyanin were reported for SAG 108.79 and ACUF 064, respectively. *G. sulphuraria* SAG 108.79 showed a protein bioaccessibility of 62%, whereas *G. sulphuraria* ACUF 064 had a protein bioaccessibility of only 14%. Regardless of these differences, both the strains displayed stable and balanced protein profiles, and promising biomass, protein, and phycocyanin production for food application [65]. Furthermore, Montenegro-Herrera et al. [66] conducted comparative studies on five strains of *G. sulphuraria* to use as a food source because of their high resilience and identified strain CCMEE 5587.1 as the best with a dry weight of 2.33 g/L in 20 days (productivity of 110 mg/L/d), protein content (44% *w*/*w*), essential amino acids (42.7% *w*/*w*), phycocyanin (4.7% *w*/*w*), total carbohydrates (5.9% *w*/*w*), and total lipids (14.1% *w*/*w*). The authors claimed this biomass to be suitable for food purposes.

## 3. Nutrient Removal

It was reported that *G. sulphuraria* can utilise contaminants and decrease their load in wastewater because of its tolerance, showing promising potential in wastewater treatment activities [67].

Primary effluent that did not undergo any biological treatment collected from the Las Cruces (USA) Wastewater Treatment Plant was used in a continuous fed-batch operation system as a growing medium for *G. sulphuraria* to study its potential for removing pollutants [68]. The authors claimed that *G. sulphuraria* decreased biochemical oxygen demand over 5 days (BOD_5_), as well as ammoniacal nitrogen and phosphates at the rate of 16.5 mg/L/day, 6.1 mg/L/day, and 1.4 mg/L/day, respectively, within three days of a fed-batch cycle time. In addition, the study by Henkanatte-Gedera et al. [69] demonstrated the potential of *G. sulphuraria* in treating urban wastewater. A single-step process based on mixotrophic metabolism exhibited its capability to reduce biochemical oxygen, nitrogen, and phosphorus demand at rates of 14.93, 7.23, and 1.38 mg/L/day, respectively.

A method that aimed for simultaneous lower-cost NH_4_^+^ removal from industrial wastewater and biomass production was demonstrated using *G. sulphuraria* photo-fermentation [70]. High protein (71.66% DW) was obtained with optimal conditions in the shake flasks system in a 4-day culture. Non-sterile fed-batch cultures in 5 L photo-fermenters resulted in a 98% NH_4_^+^ removal efficiency (removal rate of 1705.67 mg/L/d), biomass concentration of 64.65 g/L, and protein productivity of 8.75 g/L/d. The authors claimed that this method is best for highly efficient ammonium removal with a lower cost, coupled with protein-rich biomass production in an environmentally friendly way. In the same way, the potential of *G. sulphuraria* to treat swine wastewater was studied [71]. The authors indicated that regardless of high ammonium levels, a maximum growth rate of 0.72 g/L/d with the addition of 15 g/L glucose was obtained. Furthermore, a COD removal efficiency and ammonium removal rate of 94.8% and 550.5 mg/L/d, respectively, were reported. In contrast, adding glucose affects biomass composition, reducing lipid, protein, and ash contents while increasing carbohydrates. Furthermore, *G. sulphuraria* was mixotrophically cultured in urban wastewater to assess its potential for removing BOD and nutrients. The average volumetric removal rates and efficiencies were given as NH_3_-N (4.14 ± 0.94 mg/L/d and 78.9%), PO_4_ (0.82 ± 0.34 mg/L/d and 83.0%), and BOD (8.19 ± 1.95 mg/L/d and 44.8%) [72].

The capacity of *G. sulphuraria* to treat industrial effluents rich in NH_4_^+^ while producing high-protein biomass was investigated. Under sterile conditions with repeated fed-batch cultures in 5 L photo-fermenters, the maximum NH_4_^+^ recovery rate achieved was 2.19 g/L/d, with a biomass production of 55 g/L and a productivity of 12 g/L/d, comprising high contents of protein (47.6% dry weight). However, in non-sterile cultures, although the NH_4_^+^ recovery (1.79 g/L/d) and biomass production (49.5 g/L) were lower, a higher protein production of 25.3 g/L was reported [73]. Additionally, to assess the nitrogen and phosphate removal potential of *G. sulphuraria*, it was cultivated in primary settled urban wastewater. The results indicated that it could eliminate 4.7–5 mg/L/d of nitrogen and 1.5–1.7 mg/L/d of phosphate from the wastewater [74]. Moreover, another study reported that *G. sulphuraria* removed 88.3% of ammoniacal nitrogen (removal rate of 4.85 mg/L/d) and 95.5% of phosphates (1.21 mg/L/d) from urban wastewater over seven days [75].

*G. sulphuraria* under field or outdoor conditions in a 700 L photobioreactor fed with primary settled urban wastewater achieved organic carbon removal efficiency (measured as BOD_5_), ammoniacal nitrogen, and phosphate levels that ranged from 46 to 72%; 63 to 89%; and 71 to 95%, respectively [76]. Furthermore, Jiang et al. [77] reported that *G. sulphuraria* reduced 61%, 53%, and 86% of ammonia, phosphate, and BOD_5_ in primary wastewater effluent. In the same manner, Pan et al. [78] also demonstrated the potential of *G. sulphuraria* in treating raw landfill leachate, with removal efficiencies and rates for ammoniacal nitrogen and phosphate of 99.9% (22.78 mg/L/d) and 34% (2.91 mg/L/d), respectively. In another study, *G. sulphuraria* showed its potential to remove nitrogen (99.6 ± 0.2%) and phosphorus (74.2 ± 8.5%) from produced water, which describes a waste stream generated by the oil and gas industry [79]. Similarly, *G. sulphuraria* also proved its potential to remove up to 40 mg/L/d nitrogen from municipal landfill leachate [80]. According to Nirmalakhandan et al. [81], *G. sulphuraria* in a mixotrophic system again showed rates of BOD_5,_ ammoniacal nitrogen and phosphates removal from urban wastewater of 16.5 ± 3.6 mg/L/d, 6.09 ± 0.92 mg/L/d, and 1.40 ± 0.57 mg/L/d), respectively. Moreover, *G. sulphuraria* SAG 21.92, using fruit-salad production wastewater as a growing medium in shake flask cultivation at pH 2 and 42 °C, was able to show maximum specific growth (1.53 ± 0.09/d) and substrate consumption rates (2.41 ± 0.14 gSub/gDW/day), and this strain was proposed to be used in phycocyanin production [82].

## 4. Recovery of Metals

Several research results have revealed that *G. sulphuraria* can remove heavy metals from wastewater sources. It was, for instance, capable of selectively recovering over 90% of gold and palladium from aqua regia-based metal wastewater through biosorption, showing an eco-friendly and cost-effective recovery of these metals [83]. Moreover, *G. sulphuraria* was claimed to absorb precious metals such as gold, palladium, and platinum under different acidic solution levels [84]. According to Minoda et al. [85], lyophilised cells of *G. sulphuraria* recovered palladium from 4 M acid-diluted aqua regia with <135 mg/L palladium and 6 M acid solution containing <50 mg/L palladium with greater efficiency than ion-exchange resins and activated carbons. Similarly, Adams et al. [86] investigated *G. sulphuraria*’s potential in recovering gold and palladium, demonstrating high and selective adsorption capacity without modification or extensive pre-treatment, which is suitable for mass production. In an acidic environment, *Galdieria* sp. demonstrated its potential to sustainably adsorb gold and palladium from metal solutions. It showed a protein-rich cell that contains beneficial metabolites and has the potential for reduced carbon dioxide emissions, displaying commercial opportunities for eco-friendly metal recovery [87]. Fukuda et al. [88] also investigated the behaviour of *G. sulphuraria* in the presence of 30 μg/L of caesium in a potassium-deficient medium. Over 10 days, the alga exhibited a recovery rate of 52 ± 15% of the caesium present.

According to Kharel et al. [67], *G. sulphuraria* CCMEE 5587.1 achieved cadmium and lead removal efficiencies and sorption capacities of 49.80% (1.45 mg/g) and 25.10% (0.53 mg/g), respectively, in cadmium and lead ions at different concentrations (0–5 mg/L). Lyophilised cells of *G. sulphuraria* were claimed to have recovered over 90% of platinum from 2 M hydrochloric acid that contained 10 mg/L of platinum, which is comparable to the acid and platinum concentrations found in metal wastewater [89]. Similarly, *G. sulphuraria* absorbed copper from aquatic medium using stripping voltammetry [90]. Furthermore, an adsorption capacity of 35 ± 2.5 mg/g gold of lyophilised *G. sulphuraria* cells from simulated gold-containing wastewater was reported [91].

According to Ostroumov et al. [92], a significant decrease in copper (>95%) and lead (84%), followed by nickel (81.4%) and cadmium (76.7%), was obtained in an experiment where *G. sulphuraria* was used to treat laboratory-produced aqueous medium containing these metals. Employing lyophilised cells of *G. sulphuraria*, selective recovery of iridium and iron from 0.2 M hydrochloric acid with over 90% recovery efficiency was reported [93].

A study by Jalali et al. [94] showed that *G. sulphuraria* SBU-SH1 KY651246 was used to clean metal pollutants from contaminated effluent during uranium ore mines processing or in sludge resulting from pure UO_2_ processing, and they reported an excellent affinity toward the metal ions (titanium > vanadium > uranium), indicating *Galdieria*’s biosorption capabilities and favourable efficiencies. In their study, [95] indicated that *G. sulphuraria* can remove negatively charged platinum complex (PtCl_6_^2−^) at different initial concentrations (0–45 ppm), with removal efficiencies of 94.58%, 95.52%, 95.92%, and 71.81% for 10, 20, 30, and 45 ppm of PtCl_6_^2−^, respectively. In another study by Kelly et al. [96], *G. sulphuraria* was reported to transform mercuric ion (HgII) into beta-mercuric sulphide β-HgS, with a 90% efficiency.

## 5. Recovery of Rare Earth Elements

Rare earth elements (REEs) are essential materials for high-tech industries [97], and their demand is increasing [98,99]. However, acquiring them from natural resources is often challenging, indicating the need to develop efficient and environmentally friendly recycling methods [16].

*G. sulphuraria* was employed to recover REEs comprising lanthanum (36 μM), neodymium (35 μM), and dysprosium (31 μM) from solutions containing ≤15 ppm REEs. The authors reported that biosorption was poor at pH 1.5–2.5 but significantly increased (24-fold) when the pH was raised to 5 in phosphate-free conditions [100]. In the same way, greater Ln^3+^ biosorption ranging from 80 μmol/g to 130 μmol/g dry weight in a pH interval of 5–6 was obtained when investigating the potential of lifeless cells of *G. sulphuraria* to recover lanthanides from aqueous solutions [101].

According to Minoda et al. [97], greater than 90% efficiency of recovery of rare earth elements (neodymium, dysprosium, lanthanum) and copper by *G. sulphuraria* from a solution containing 0.5 ppm was reported. Furthermore, Palmieri et al. [16] investigated the potential of freeze-dried cells of *G. sulphuraria* to recover rare earth elements (yttrium, cerium, europium, and terbium) from quaternary-metal aqueous solutions and reported that all rare earth elements were absorbed efficiently at pH 4.5, with the lowest dose of biosorbent in 30 min. However, a higher removal rate of cerium was obtained at pH 2.5 after 360 min.

Living cells of *G. sulphuraria* were employed to recover four rare earth elements, yttrium, cerium, europium, and terbium, from single- and quaternary-metal aqueous solutions, using two different strains, SAG 107.79 and ACUF 427, at varying pH levels. The result showed that all rare earth elements were recovered, but removal performances were strain- and pH-dependent for all metal ions [98]. Similarly, the growth performance and rare earth elements (cerium, neodymium, Lanthanum, yttrium) recovery potential of *G. sulphuraria* in red mud (a by-product of the production of alumina from bauxite ore) was studied [102]. The authors revealed suppressed growth, stable photosynthetic performance, and rare earth recovery with a higher accumulation (109 μg/g DM) of rare earth elements in mixotrophic than autotrophic culture. Additionally, Iovinella et al. [103] used the freeze-dried biomass of *G. sulphuraria* to recover rare earth elements (yttrium, europium, cerium, gadolinium, terbium, and lanthanum) from spent fluorescent lamp (FL) luminophores and reported high biosorption of yttrium (287.42 mg/g DM, 91.60% of all REEs) and europium (20.98 mg/g, 6.69%) with 5 mg/mL biomass dosage after 5 min, but cerium, gadolinium, terbium, and lanthanum were in trace amounts. Similarly, Singh et al. [99] studied the potential of *G. sulphuraria* to recover rare earth elements from compact fluorescent lamps (CFLs). They reported that specific rare earth elements accumulated differently at different cell-cycle phases: yttrium, europium, lanthanum, and cerium were the abundant lanthanides accumulated by *G. sulphuraria*. Moreover, removal efficiencies of 97.19%, 96.19%, and 98.87% for Lanthanum, yttrium, and samarium, respectively, were reported from acidic rare earth mining wastewater, using calcium alginate-immobilised *G. sulphuraria* beads [104].

## 6. Pathogen Reduction

*G. sulphuraria* has also demonstrated its potential to reduce different pathogens in wastewater. For instance, Tchinda et al. [68] used primary effluent in a continuous fed-batch operation system as a growing medium of *G. sulphuraria* and reported non-detectable levels (<1 CFU/100 mL) of total coliform (a group of bacteria) and faecal coliform (a subgroup of coliform bacteria) counts within three days. Moreover, Delanka-Pedige et al. [105] compared the results of a *G. sulphuraria* wastewater treatment system without any chlorination with a conventional wastewater treatment system with chlorination. They reported that log removal of somatic coliphages (3.13 ± 0.34), F-specific coliphages (1.23 ± 0.34), enterovirus (1.05 ± 0.32), and norovirus GI (1.49 ± 0.16) were comparable. At the same time, there was a diverse (250 species) virus community in the chlorinated effluent of the conventional system, but there were only 14 discrete virus species, that are even not pathogenic to humans, in the un-chlorinated effluent of the algal-based system. In a similar study, a reduction of 3.3 log units of the total coliform in the influent (2.3 × 107 CFU/100 mL) in a wastewater treatment system was reported, but no total or faecal coliform was detected at all in the *G. sulphuraria* effluent. Moreover, qPCR analysis confirmed 98% removal of total bacteria and complete removal of *Enterococcus faecalis* and *Escherichia coli* in the algal system [106]. Additionally, *G. sulphuraria*-based wastewater treatment and conventional wastewater treatment systems fed with the same primary effluent were reported to have shown different results, whereby the algal system reduced concentrations of antibiotic (erythromycin and sulfamethoxazole)-resistant bacteria in the effluent more effectively than the conventional treatment system. In addition, the algal system reduced more of the relative abundance of antibiotic resistance genes, qnrA, qnrS, tetW and intI1, in the surviving bacteria, which were increased in the conventional wastewater treatment system [107]. Furthermore, a comparison between an algal-based wastewater treatment system that employed *G. sulphuraria* and a conventional activated sludge-based (CAS) wastewater treatment system revealed that all classes of ARGs (antibiotic-resistant genes) and VGs (virulence genes) were reduced in their relative abundance in the algal wastewater treatment system [108].

According to Pleissner et al. [109], no pathogen, such as Salmonella sp., could be detected in a non-sterile fed-batch culture of *G. sulphuraria* in wastewater from a fish processing facility, slam (a mix of used fish feed and faeces), and dried pellet (sediments from enzymatic hydrolysis of rainbow trout). In assessing the potential of *G. sulphuraria* as a possible producer of bioactive compounds with antiviral activity against herpesviruses and coronaviruses, the algal extract displayed intense antiviral activity at non-toxic concentrations against all tested enveloped viruses [110].

Similarly, Pleissner et al. [111] investigated digestate as a nitrogen source when cultivating *G. sulphuraria* and reported a diminishing of *Salmonella* sp., yeast, and moulds, Enterobacteriaceae, as well as Enterococci within 24 h of hydrolysis or cultivation. In addition, the counts of aerobic and mesophilic organisms were subsequently reduced by a log reduction factor of 3, and spore-forming microorganisms were reduced by a log reduction factor of 2 during cultivation under acidic conditions.

The efficacy of *G. sulphuraria* in reducing pathogen levels in wastewater is demonstrated in the findings of the above studies. The synthesis of bioactive compounds with antiviral and antimicrobial properties against herpesviruses and coronaviruses was studied. However, most of the reports did not list the reason for the reduction in pathogens. Further studies on the cause of pathogen reduction are required, as this could result from the algal physiological mechanisms or environmental conditions such as low pH levels (2.5–3), which showed, for example, coliform inactivation compared to the higher pH values [112].

## 7. Conclusions and Future Research Directions

This manuscript documented research results obtained using the thermo-acidophilic microalga *G. sulphuraria*, which revealed its immense potential applications in different biotechnological disciplines. It has been reported that *G. sulphuraria* can synthesise bioactive compounds, such as phycocyanin, that have a broad range of application areas, such as in the cosmetics and food industries. It also demonstrated antioxidant properties that are a massive gain in combating health-related issues. In addition, its enormous potential to contribute to environmental remediation is impressive. It can remove nutrients and heavy metals and reduce pathogens from several wastewater sources. Moreover, it has the potential to recover rare earth elements sustainably. Future research on *G. sulphuraria* should focus on different areas to fully exploit its potential. Optimisation of its cultivation condition is a point that should be investigated further. In addition, as observed from this review, most of the research results obtained using *G. sulphuraria* are at a laboratory scale under a controlled environment. More outdoor or field-level trials are needed as a first move towards commercial-scale production. Integrating *G. sulphuraria* in wastewater treatment facilities is another issue that needs due consideration to help efficient nutrient removal. *Galdieria*’s potential to produce nutritional profiles, such as protein, carbohydrates, and lipids will benefit from further investigations, as these could serve as sustainable food ingredients in, for example, aquaculture. The contribution of this alga to sustainable development and environmental well-being could be achieved by implementing the above points.

## Data Availability

No new data were created or analysed in this study. Data sharing does not apply to this article.
